# Extracting accurate light–matter couplings from disordered polaritons

**DOI:** 10.1515/nanoph-2024-0049

**Published:** 2024-04-15

**Authors:** Kai Schwennicke, Noel C. Giebink, Joel Yuen-Zhou

**Affiliations:** Department of Chemistry and Biochemistry, 8784 University of California San Diego, La Jolla, CA 92093, USA; Department of Electrical Engineering and Computer Science, and Physics, 1259 University of Michigan, Ann Arbor, MI 48109, USA

**Keywords:** molecular polaritons, strong light-matter coupling, disorder

## Abstract

The vacuum Rabi splitting (VRS) in molecular polaritons stands as a fundamental measure of collective light–matter coupling. Despite its significance, the impact of molecular disorder on VRS is not fully understood yet. This study delves into the complexities of VRS amidst various distributions and degrees of disorder. Our analysis provides precise analytical expressions for linear absorption, transmission, and reflection spectra, along with a “sum” rule, offering a straightforward protocol for extracting accurate collective light–matter coupling values from experimental data. Importantly, our study cautions against directly translating large VRS to the onset of ultrastrong coupling regime. Furthermore, for rectangular disorder, we witness the emergence of narrow side bands alongside a broad central peak, indicating an extended coherence lifetime even in the presence of substantial disorder. These findings not only enhance our understanding of VRS in disordered molecular systems but also open avenues for achieving prolonged coherence lifetimes between the cavity and molecules via the interplay of collective coupling and disorder.

## 1 Introduction

In the realm of molecular polaritons, the phenomenon of vacuum Rabi splitting (VRS) stands as an established metric for gauging the strength of collective light–matter coupling. Traditionally, this interaction is classified into several regimes: weak, strong, ultrastrong, and deep-strong [1]. In particular, molecular polaritons are often observed in the realm of strong coupling, with a wide range of potential applications such as catalysis [2], [3], [4], [5], exciton transport [6], [7], [8], [9], [10], [11], and Bose–Einstein condensation [12], [13], [14]. While reaching the ultrastrong coupling regime remains experimentally challenging, there are a number of experiments that have pushed the limits of molecular systems into this intriguing regime [15], [16], [17], [18].

**Figure 1:**
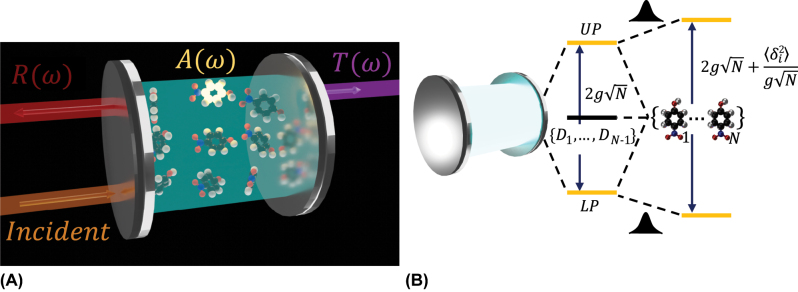
Linear response of molecular polaritons in the presence of molecular disorder. (A) Linear spectroscopy of molecular polaritons as absorption *A*(*ω*), transmission *T*(*ω*), and reflection *R*(*ω*). (B) For weak disorder, a perturbative approach to understand the role of disorder is useful. The zeroth-order Hamiltonian can be taken to be the disorderless system, comprising of the photon mode interacting with the *N* degenerate molecules to form the upper (UP) and lower (LP) polaritons, alongside *N* − 1 dark states ({*D*_1_, …, *D*_*N*−1_}). The VRS at resonance in this case happens to be $2g\sqrt{N}$ , where *g* is the single molecule light–matter coupling. The molecular disorder then perturbatively couples the polaritons to the manifold of dark states, inducing level repulsion between the polaritons, as discussed in Refs. [35], [38], [39], thus increasing the VRS. This repulsion depends on the variance of the disorder distribution ( $\left\langle \delta_{i}^{2}\right\rangle$ , where *δ*_*i*_ = *ω*_*i*_ − *ω*_0_ and *ω*_0_ represents the center of the distribution). Given the perturbative character of this analysis, it does not apply to strong disorder.

In the idealized scenario of *N* identical molecules strongly coupled to a single photonic mode, the magnitude of VRS scales linearly with $\sqrt{N}$ [19], [20] (see Figure 1). However, the inherent complexity of molecular ensembles introduces a compelling challenge, as molecular disorder becomes an inescapable feature. Molecular disorder exerts a profound influence on various aspects of polariton physics, including transport [7], [8], [10], [21], [22], [23], [24], [25], photoconductivity [26], photoreactivity [27], and vibropolaritonic chemistry [28]. In the solid-state physics community, using polaritons to reduce the linewidth of quantum emitters has potential applications in the storage of quantum information [29], [30]. Surprisingly, even though the effects of disorder where theoretically studied early on Refs. [31], [32], [33], [34], the effects of disorder on VRS splitting are still a debate within the community. Early explorations by Houdré et al. [33] suggested that disorder (or inhomogeneous broadening) should have no impact on the size of the splitting. However, these conclusions have been contested in recent investigations [8], [35], [36], [37], [38], [39], [40] which note that disorder can both enhance and suppress the VRS.

In this article, we revisit the problem of VRS and disorder, embarking on a comprehensive study of linear absorption *A*, transmission *T*, and reflection *R* properties of molecular polaritons, considering various distributions and magnitudes of disorder. Our aim is not only to elucidate the intricate interplay between molecular disorder and VRS, but also to provide a robust method for accurately extracting light–matter coupling parameters. Our findings align with recent reports, demonstrating that VRS tends to increase with disorder, reaches a saturation point, and eventually decreases to zero for a wide range of disorder distributions. Note that *A*, *T*, *R* do not in general give the same value of VRS [32]. Hence, while some of the results are already known in the literature, we deem it valuable to collect all the results in a single study. Significantly for experiments, we unveil what seems to be a ubiquitous scenario: the presence of substantial disorder can dramatically enhance the VRS leading to an *apparent* onset of the ultrastrong coupling regime, despite the underlying collective light–matter coupling firmly residing within the strong coupling regime. Moreover, we introduce a novel sum rule that proves instrumental in extracting precise values of collective light–matter coupling, particularly when both absorption and transmission can be measured in the experimental setup. Crucially, this sum rule demonstrates generality across all types of disorder. In the case of a rectangular distribution (which was briefly discussed in Ref. [36]), we observe the emergence of two narrow polariton peaks in the spectra, reminiscent of the pronounced spectral narrowing witnessed in the context of surface lattice resonances [41] and other phenomena associated with Wood anomalies [42], [43], [44], [45], [46].

## 2 Results

### 2.1 Model

For concreteness, we consider *N* two level systems coupled to a single photon mode (in the rotating wave approximation [RWA], Tavis–Cummings model [19]):

(1) $$H=\hbar \omega_{ph}a^{†}a+\sum_{i=1}^{N}\hbar \omega_{ex,i}\sigma_{i}^{†}\sigma_{i}-\hbar \lambda \left(\sum_{i=1}^{N}\mu_{i}a\sigma_{i}^{†}+\text{h.c.}\right),$$

where *ω*_ph_ and *a* are the photon frequency and annihilation operator, *ω*_ex,*i*_ and *σ*_*i*_ = |*g*_*i*_⟩⟨*e*_*i*_| are the frequency and annihilation operator for the *i*th two level system, *μ*_*i*_ is the amplitude of the *i* transition dipole, and $\hbar \lambda =\sqrt{\frac{\hbar \omega_{ph}}{2\epsilon_{0}V_{ph}}}$ is the vacuum field amplitude where *ϵ*_0_ is the vacuum permittivity and $V_{ph}$ is the cavity mode volume. In the thermodynamic limit (*N* → ∞), the linear absorption, transmission, and reflection spectra are given by (see Refs. [47], [48])

(2) $$A\left(\omega \right)=\frac{\kappa_{L}\omega_{ph}\chi^{\prime \prime }\left(\omega \right)}{|\omega -\omega_{ph}+i\frac{\kappa }{2}+\frac{\omega_{ph}}{2}[\chi^{\prime }\left(\omega \right)+i\chi^{\prime \prime }\left(\omega \right)]{|}^{2}},$$

(3) $$T\left(\omega \right)=\frac{\kappa_{L}\kappa_{R}}{|\omega -\omega_{ph}+i\frac{\kappa }{2}+\frac{\omega_{ph}}{2}[\chi^{\prime }\left(\omega \right)+i\chi^{\prime \prime }\left(\omega \right)]{|}^{2}},$$

(4) R(ω)=1−A(ω)−T(ω).

Note, for simplicity we are considering the case where the volume of the molecular sample is equal to the cavity mode volume (*i.e.* $V_{\mathrm{mol}}=V_{\mathrm{ph}}$ ). Here *κ* = *κ*_*L*_ + *κ*_*R*_ is the total cavity decay rate, and the respective decay rates into the left and right photon continua are denoted by *κ*_*L*_ and *κ*_*R*_. The linear molecular susceptibility *χ*(*ω*) is given by

(5) $$\chi \left(\omega \right)=-\lim_{\gamma \to 0^{+}}\frac{1}{\hbar \varepsilon_{0}V_{mol}}\sum_{i}^{N}\tanh\left(\frac{\hbar \omega_{\text{ex},i}}{2k_{B}T}\right)\frac{|\mu_{i}{|}^{2}}{\omega -\omega_{\text{ex},i}+i\frac{\gamma }{2}}$$

Considering the case when *ℏω*_ex,*i*_ ≫ *k*_*B*_ *T* and assuming that all *N* two level systems have the same transition-dipole amplitude *μ*, the molecular susceptibility, multipled by a factor $\frac{\omega_{\mathrm{ph}}}{2}$ , becomes

(6) $$\begin{array}{ll} \frac{\omega_{ph}}{2}\chi \left(\omega \right) & =-\lim_{\gamma \to 0^{+}}g^{2}N\int d\omega_{\text{ex}}\frac{p\left(\omega_{\text{ex}}\right)}{\omega -\omega_{\text{ex}}+i\frac{\gamma }{2}}\\ & =\frac{\omega_{ph}}{2}[\chi^{\prime }\left(\omega \right)+i\chi^{\prime \prime }\left(\omega \right)], \end{array}$$

where *g* ^2^ = |*λμ*|^2^ is the square of the single molecule light–matter coupling, *p*(*ω*_ex_) is the probability distribution of excitation frequencies, and the real and imaginary parts of the molecular susceptibility, multipled by a factor $\frac{\omega_{\mathrm{ph}}}{2}$ , are $\frac{\omega_{ph}}{2}\chi^{\prime }\left(\omega \right)=-g^{2}NP\int d\omega_{\text{ex}}\frac{p\left(\omega_{\text{ex}}\right)}{\omega -\omega_{\text{ex}}}$ , where P is the Cauchy principal value, and $\frac{\omega_{\mathrm{ph}}}{2}$ *χ*″(*ω*) = *g* ^2^ *Nπp*(*ω*). It is insightful to note that the simple dependence of the polariton absorption spectrum on the bare molecular linear susceptibility is similar to the simple dependence of the spectroscopic response of molecular aggregates, under the coherent potential approximation, on the response of the isolated monomeric units [49].

To explore the effects of *p*(*ω*_ex_), we consider Lorentzian,

(7) $$p\left(\omega_{\text{ex}}\right)=\frac{1}{\pi }\frac{\sigma /2}{{\left(\omega_{\text{ex}}-\omega_{0}\right)}^{2}+{\left(\sigma /2\right)}^{2}},$$

Gaussian,

(8) $$p\left(\omega_{\text{ex}}\right)=\frac{1}{\sqrt{2\pi }\sigma }e^{-\frac{1}{2}{\left(\frac{\omega_{\text{ex}}-\omega_{0}}{\sigma }\right)}^{2}},$$

and rectangular

(9) $$p\left(\omega_{\text{ex}}\right)=\frac{1}{\sigma }\text{rec}\left[2\left(\omega_{\text{ex}}-\omega_{0}\right)/\sigma \right],$$

(10) $$\text{rec}\left[y\right]=\left\{\begin{array}{cc} 1,\; & |y|\leq 1\\ 0,\; & |y|>1 \end{array}\right.$$

disorder. Table 1 lists the analytical expressions of *χ*′(*ω*) and *χ*″(*ω*) for the three different distributions.

**Table 1:** Real and imaginary parts of the molecular susceptibility.

Real or imaginary	Susceptibility		
	Gaussian^a^	Lorentzian	Rectangle
$\frac{\omega_{\mathrm{ph}}}{2}$ *χ*′(*ω*)	$-\frac{g^{2}N\sqrt{2}}{\sigma }F\left(\frac{\omega -\omega_{0}}{\sqrt{2}\sigma }\right)$	$-g^{2}N\frac{\left(\omega -\omega_{0}\right)}{{\left(\omega -\omega_{0}\right)}^{2}+{\left(\sigma /2\right)}^{2}}$	$-\frac{g^{2}N}{\sigma }\ln|\frac{\omega -\omega_{0}+\sigma /2}{\omega -\omega_{0}-\sigma /2}|$
$\frac{\omega_{\mathrm{ph}}}{2}$ *χ*″(*ω*)	$\frac{g^{2}N}{\sigma }\sqrt{\frac{\pi }{2}}\exp\left[-\frac{1}{2}{\left(\frac{\omega -\omega_{0}}{\sigma }\right)}^{2}\right]$	$g^{2}N\frac{\sigma /2}{{\left(\omega -\omega_{0}\right)}^{2}+{\left(\sigma /2\right)}^{2}}$	$\frac{g^{2}N\pi }{\sigma }\text{rec}\left[2\left(\omega -\omega_{0}\right)/\sigma \right]$

^a^Here $F\left(y\right)=\exp\left[-y^{2}\right]\int_{0}^{y}\text{d}te^{t^{2}}$ is the Dawson function.

### 2.2 Lorentzian disorder

For Lorentzian disorder, the real and imaginary parts of the susceptibility are

(11) $$\frac{\omega_{ph}}{2}\chi^{\prime }\left(\omega \right)=-g^{2}N\frac{\left(\omega -\omega_{0}\right)}{{\left(\omega -\omega_{0}\right)}^{2}+{\left(\sigma /2\right)}^{2}},$$

(12) $$\frac{\omega_{ph}}{2}\chi^{\prime \prime }\left(\omega \right)=g^{2}N\frac{\sigma /2}{{\left(\omega -\omega_{0}\right)}^{2}+{\left(\sigma /2\right)}^{2}}.$$

From Eqs. (2) and (3), the absorption, transmission, and reflection spectra are given by

(13) $$A\left(\omega \right)=\frac{\kappa_{L}\sigma g^{2}N}{|\left(\omega -\omega_{\text{ph}}+i\frac{\kappa }{2}\right)\left(\omega -\omega_{0}+i\frac{\sigma }{2}\right)-g^{2}N{|}^{2}},$$

(14) $$T\left(\omega \right)=\frac{\kappa_{L}\kappa_{R}\left[{\left(\omega -\omega_{0}\right)}^{2}+{\left(\sigma /2\right)}^{2}\right]}{|\left(\omega -\omega_{\text{ph}}+i\frac{\kappa }{2}\right)\left(\omega -\omega_{0}+i\frac{\sigma }{2}\right)-g^{2}N{|}^{2}},$$

(15) $$R\left(\omega \right)=1-\frac{\kappa_{L}\left\{\kappa_{R}\left[{\left(\omega -\omega_{0}\right)}^{2}+{\left(\sigma /2\right)}^{2}\right]+\sigma g^{2}N\right\}}{|\left(\omega -\omega_{\text{ph}}+i\frac{\kappa }{2}\right)\left(\omega -\omega_{0}+i\frac{\sigma }{2}\right)-g^{2}N{|}^{2}}.$$

**Figure 2:**
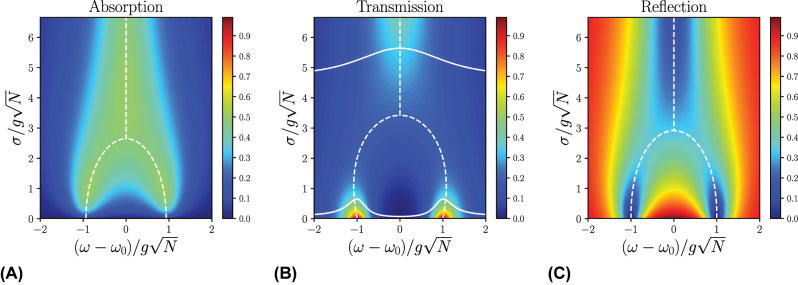
Numerically calculated (A) absorption, (B) transmission, and (C) reflection spectra for a Lorentzian distribution of excitation energies *p*(*ω*_ex_) centered at *ω*_ex_ = *ω*_0_; *ω*_ph_ = *ω*_0_ and $\kappa_{L}=\kappa_{R}=\frac{1}{2}g\sqrt{N}$ . The white dashed lines indicate our analytical results for the polariton frequencies, showing strong agreement with the calculated spectra overall magnitudes of disorder. As done in Ref. [35], the white solid lines in (B) represent individual spectra for weak and strong disorder systems, highlighting the transition from two distinct peaks to a broad central peak as disorder increases.

Figure 2 presents the numerically calculated spectra.

To find the extrema of each spectra, we solve for $\frac{\text{d}}{\text{d}\omega }T\left(\omega \right)=0$ , $\frac{\text{d}}{\text{d}\omega }A\left(\omega \right)=0$ , and $\frac{\text{d}}{\text{d}\omega }R\left(\omega \right)=0$ separately. Lorentzian disorder affords exact analytical expressions for all regimes of *σ*. In the case where the photon mode is resonant with the center of the Lorentzian distribution, *i.e.*, *ω*_ph_ = *ω*_0_, we find that the upper $\left(\omega_{+}^{A}\right)$ and lower $\left(\omega_{-}^{A}\right)$ polariton peaks in the absorption are located at the frequencies

(16) $$\omega_{\pm }^{A}=\omega_{0}\pm I\left[\sqrt{\frac{1}{8}\left(\sigma^{2}+\kappa^{2}\right)-g^{2}N}\right].$$

Similarly, the upper and lower polariton peaks in the transmission spectrum are located at

(17) $$\omega_{\pm }^{T}=\omega_{0}\pm I\left[\sqrt{\frac{\sigma^{2}}{4}-g^{2}N\sqrt{1+\frac{\sigma \left(\kappa +\sigma \right)}{2g^{2}N}}}\right],$$

and the peaks in the reflection spectrum are at

(18) $$\begin{array}{ll} \omega_{\pm }^{R} & =\omega_{0}\pm I\left[\left(\frac{\sigma^{2}}{4}+\frac{\sigma g^{2}N}{\kappa_{R}}-g^{2}N\right.\right.\\ & \;\left.{\left.\times \sqrt{\frac{\sigma \left(\kappa +\sigma \right)\left(\sigma -\kappa +2\kappa_{R}\right)}{4\kappa_{R}g^{2}N}+{\left(1+\frac{\sigma }{\kappa_{R}}\right)}^{2}}\right)}^{1/2}\right]. \end{array}$$

With prior information on the molecular disorder (*σ*) and cavity linewidth (*κ*), one can use the above equations to extract the correct value for the collective coupling $g\sqrt{N}$ from experimentally obtained spectra. For the transmission spectra, we observe similar behavior to that was shown in Refs. [35], [36], [37], [38], [39] for Gaussian disorder: the VRS initially increases with increasing disorder; as disorder increases further, the VRS saturates, and then decreases to zero. It is interesting that this trend is not observed for absorption, as VRS only *decreases* with disorder. Table 2 summarizes the intricate behavior of VRS across spectra for different disorder distributions. Qualitatively, the increase of VRS with weak disorder is a manifestation of level repulsion (see Figure 1B). Additionally, the collapse of the VRS and the transition from two peaks to a single central peak can be related to an exceptional point pertaining to the complex valued pole of the polariton transmission spectrum [8]. It should be noted that Eqs. (13) and (14) are the same as those presented in Ref. [32]. Furthermore, the expressions for *κ* = 0, Eq. (16) looks similar to the analytical expressions derived by Refs. [8], [36], [40], but the decrease in VRS for weak disorder differs by a factor of a half compared to our result. This is due to the fact that the real parts of the poles for Eqs. (16)–(18) do not correspond to the true extrema along the real-value frequency axis.

**Table 2:** Vacuum Rabi splitting trends with disorder.

Optical signal	Gaussian		Lorentzian		Rectangle	
	$\sigma <g\sqrt{N}$	$\sigma >g\sqrt{N}$	$\sigma <g\sqrt{N}$	$\sigma >g\sqrt{N}$	$\sigma <g\sqrt{N}$	$\sigma >g\sqrt{N}$
*A*(*ω*)	Increases	Decreases	Decreases	Decreases	Increases	Decreases; narrow side bands
*T*(*ω*)	Increases	Decreases	Increases	Decreases	Increases	Decreases; narrow side bands
*R*(*ω*)	Increases	Decreases	Increases or decreases^a^	Decreases	Increases	Decreases; narrow side bands

^a^For the Lorenzian, the VRS in the reflection spectrum increases if $\kappa_{L}^{2}/\kappa_{R}^{2}<1$ and decreases if $\kappa_{L}^{2}/\kappa_{R}^{2}>1$ (see Table 3).

### 2.3 Gaussian and rectangular disorder

For Gaussian and Rectangular disorder, we can still extract semi-analytical results when $\sigma \ll g\sqrt{N}$ (weak disorder) and $|\omega -\omega_{0}|\approx g\sqrt{N}$ (about the polariton peaks). For the Gaussian distribution, we employ the asymptotic expansion of the Dawson function [50], similar to Ref. [36], up to $O\left[{\left(\frac{\sigma }{\omega -\omega_{0}}\right)}^{3}\right]$ to obtain an approximate expression for the real part of the susceptibility, multipled by a factor $\frac{\omega_{\mathrm{ph}}}{2}$ ,

(19) $$\frac{\omega_{ph}}{2}\chi^{\prime }\left(\omega \right)\approx -g^{2}N\left[\frac{1}{\omega -\omega_{0}}+\frac{\sigma^{2}}{{\left(\omega -\omega_{0}\right)}^{3}}\right].$$

Meanwhile, for the rectangular distribution, we get,

(20) $$\frac{\omega_{ph}}{2}\chi^{\prime }\left(\omega \right)\approx -g^{2}N\left[\frac{1}{\omega -\omega_{0}}+\frac{\sigma^{2}}{12{\left(\omega -\omega_{0}\right)}^{3}}\right].$$

**Figure 3:**
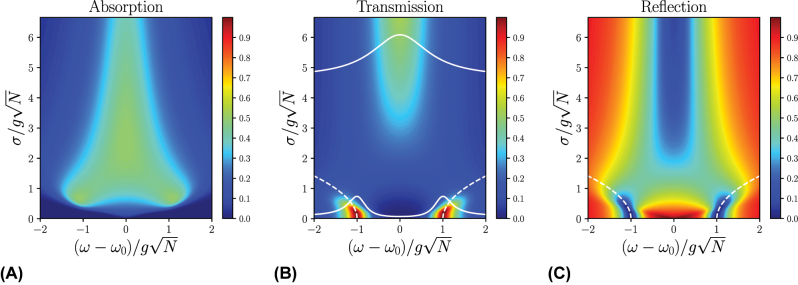
Numerically calculated (A) absorption, (B) transmission, and (C) reflection spectra for a Gaussian distribution of excitation energies *p*(*ω*_ex_) centered at *ω*_ex_ = *ω*_0_; *ω*_ph_ = *ω*_0_ and $\kappa_{L}=\kappa_{R}=\frac{1}{2}g\sqrt{N}$ . The white dashed lines indicate our analytical results for the polariton frequencies, showing strong agreement with the calculated spectra for weak disorder. The white solid lines in (B) represent individual spectra for weak and strong disorder systems, highlighting the transition from two distinct peaks to a broad central peak as disorder increases.

**Figure 4:**
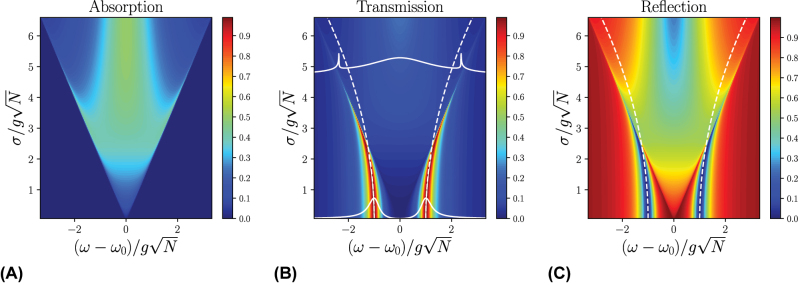
Numerically calculated (A) absorption, (B) transmission, and (C) reflection spectra for a rectangular distribution of excitation energies *p*(*ω*_ex_) centered at *ω*_ex_ = *ω*_0_; *ω*_ph_ = *ω*_0_ and $\kappa_{L}=\kappa_{R}=\frac{1}{2}g\sqrt{N}$ . The white dashed lines indicate our analytical results for the polariton frequencies, showing strong agreement with the calculated spectra for weak disorder. The white solid lines in (B) represent individual spectra for weak and strong disorder systems, highlighting the transition from two distinct peaks to a broad central peak as disorder increases. Remarkably, for large disorder, two peaks that are narrower than the line width of the cavity and molecular disorder emerge on either side of the broad central peak.

Note that for both distributions *χ*″(*ω*) ≈ 0 at the polaritonic windows, so no VRS is predicted in the absorption spectrum for both Gaussian and rectangular distributions for low disorder. The lack of VRS in the absorption spectrum in the case of low disorder for these distributions is due to the minimal overlap between the molecular absorption spectrum and the polariton transmission, since the tails of the Gaussian die quickly away from *ω*_0_, while the rectangular distribution has no tails. Contrast this observation with the analogous one for the Lorentzian distribution, which does present VRS in its absorption spectrum owing to the long tails of the Lorentzian. As disorder increases, the tails of the Gaussian and rectangular distributions overlap more with the polariton transmission peaks, and VRS begins to appear in the polariton absorption spectrum. However, as disorder becomes increasingly stronger, the VRS collapses, resulting in a broad central peak (see Figures 3A and 4A). Understanding the difference in overlap of the Lorenzian and Gaussian tails with the polariton transmission peaks can be used to engineer systems with higher polariton quality factors; for example, Ref. [51] proposes using an applied magnetic field to convert a Lorentzian disorder distribution into a Gaussian one in order to remove the influence of the Lorentzian tails.

Using these approximations, we find that for *ω*_ph_ = *ω*_0_, the transmission and reflection spectra for Gaussian distribution are approximately

(21) $$T\left(\omega \right)\approx \frac{\kappa_{L}\kappa_{R}g^{6}N^{3}}{|{\left(\omega -\omega_{0}\right)}^{4}-g^{2}N{\left(\omega -\omega_{0}\right)}^{2}-g^{2}N\sigma^{2}+ig^{3}N^{\frac{3}{2}}\frac{\kappa }{2}{|}^{2}},$$

(22) R(ω)≈1−T(ω),

with the polariton frequencies for both spectra approximately located at

(23) $$\omega_{\pm }=\omega_{0}\pm \sqrt{\frac{1}{2}g^{2}N+\frac{1}{2}\sqrt{g^{4}N^{2}+4g^{2}N\sigma^{2}}}.$$

Similarly, we find that the transmission and reflection spectra for the rectangular distribution are approximately

(24) $$T\left(\omega \right)\approx \frac{\kappa_{L}\kappa_{R}g^{6}N^{3}}{|{\left(\omega -\omega_{0}\right)}^{3}-g^{2}N\left(\omega -\omega_{0}\right)-\frac{g^{2}N\sigma^{2}}{12}+ig^{3}N^{\frac{3}{2}}\frac{\kappa }{2}{|}^{2}},$$

(25) R(ω)≈1−T(ω).

In this case the polariton frequencies are approximately located at

(26) $$\omega_{\pm }=\omega_{0}\pm \sqrt{\frac{1}{2}g^{2}N+\frac{1}{2}\sqrt{g^{4}N^{2}+\frac{\sigma^{2}g^{2}N}{3}}},$$

which is similar to Eq. (23).

Figure 3 shows the numerically calculated spectra for Gaussian disorder. The trend in VRS in the transmission and reflection spectra as a function of *σ* is qualitatively the same as that for the Lorentzian: the VRS initially increases for $\sigma <g\sqrt{N}$ , and then decreases to zero for $\sigma >g\sqrt{N}$ (see Table 2). Additionally, the initial increase in VRS for the Gaussian and rectangular distributions, for weak disorder, can be understood through the mechanism of level repulsion between the polariton states and the dark states, which are coupled by the molecular disorder. Eq. (23) is in good agreement with the numerical results for $\sigma \ll g\sqrt{N}$ , and upon Taylor expanding around *σ* = 0 to second order, reduces to the analytical results of Refs. [36], [39]. The analytical results of Refs. [35], [38] qualitatively capture the behavior of the transmission spectrum; however, quantitatively they fits better with the Lorentzian disorder. The transformation of Gaussian into Lorentzian disorder in these references appears to be due to the usage of the Markovian approximation. Figure 3 also highlights the dangers for taking VRS at face value. We observe that the largest VRS in the transmission and reflection spectra is approximately $1.5\times 2g\sqrt{N}$ . This implies that one must be careful using VRS to determine the strength of the collective light–matter coupling. For example, if VRS/2*ω*_0_ ≈ 0.1 one may mistakenly claim to be in the ultrastrong coupling regime, which is typically defined as the region where $g\sqrt{N}/\omega_{0}≳0.1$ [52], [53], while in reality the collective light–matter coupling $g\sqrt{N}/\omega_{0}\approx 0.07$ is still within the strong-coupling regime.

Figure 4 displays the numerically calculated spectra for the rectangular disorder. Such a scenario was briefly considered in Ref [36]; here, we provide further analysis. At low disorder, the rectangular distribution exhibits similar behavior to the Gaussian distribution, as predicted by Eq. (26). As disorder increases, a broad central peak forms, consistent with both the Lorentzian and Gaussian distributions. Intriguingly, as disorder increases, two sharp sidebands for the polaritons also emerge, each narrower than the cavity linewidth and the width of the rectangular distribution, respectively. This is reminiscent to the pronounced spectral narrowing witnessed in plasmonic surface lattice resonances [41]. The unique characteristics of the rectangular disorder, including singularities in its real part of the susceptibility *χ*′(*ω*) near these polariton peaks (see Table 1), are responsible for this phenomenon. The reduced linewidth of these peaks indicates a higher degree of coherence lifetime within the system, even in the presence of significant energetic disorder. Consequently, this observation presents a promising avenue for applications in molecular polariton systems requiring prolonged coherences between cavity and molecules. In a realistic experiment, this phenomenon will only occur if the underlying members of the disorder distribution have a small Lorentzian homogeneous linewidth (which has not been treated in this work); a large such linewidth will smoothen the singularity and reduce the problem to the Lorentzian case.

**Figure 5:**
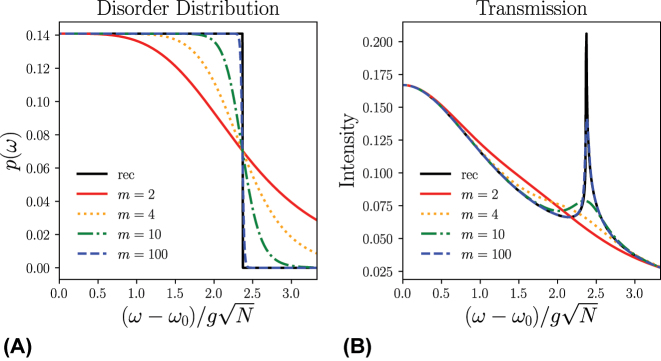
Discontinuous versus continuous disoder distribution edge. (A) Comparing the discontinuous rectangular (rec) distribution with the continuous function *R*_*m*_ for different values of *m*. As *m* increases, the edge becomes sharper, such that the continuous distribution begins to mimic the rectangular one. (B) Comparing the resulting polariton transmission spectra for large disorder where the rectangular distribution has sharp sidebands. For *m* = 2, only a broad central peak is observed, but as *m* increases the sidebands emerge and become sharper.

For completeness, we study the effects of rounding the discontinuous edge of the rectangular distribution. Consider the continuous distribution

(27) $$p_{m}\left(\omega_{ex}\right)=\frac{1}{\sigma }R_{m}\left[2\left(\omega_{ex}-\omega_{0}\right)/\sigma \right],$$

where

(28) $$R_{m}\left[y\right]=\frac{1}{y^{2m}+1}$$

and *m* is a positive integer. In the limit *m* → ∞, *R*_*m*_[*y*] mimics the rectangular function rec[*y*] (see Eq. (9)). Figure 5 shows that for small values of *m*, where the “edge” of the distribution is relatively smooth, only a single broad central peak is observed. As *m* increases, the two sidebands emerge and become sharper, as does the edge of the distribution. These results highlight that the survival of these sidebands heavily depends on the steep rise at the edge of the distribution, though this edge need not be perfect due to the fact that these spectral features are observed for finite *m* where the corners of the distribution are rounded. A candidate system would be a uniform distribution of quantum emitter frequencies, possibly engineered with quantum dots that have a narrow homogeneous linewidth.

### 2.4 Sum rule

From our extensive study of disorder effects on the polariton linear spectrum, it is evident from the three disorder distributions studied in this work, that disorder significantly impacts the value of VRS, seemingly posing a challenge in extracting the precise value of the collective light–matter coupling. However, a simple sum rule can be utilized when both the absorption and transmission polariton spectra are accessible. By integrating the ratio of these two signals (*I*), which is proportional to the imaginary part of the linear molecular susceptibility multiplied by a factor $\frac{\omega_{\mathrm{ph}}}{2}$ , *χ*″(*ω*) = *g* ^2^ *Nπp*(*ω*), a robust method for determining the collective light–matter coupling is revealed (see Eqs. (2) and (3)):

(29) $$I=\int d\omega \frac{A\left(\omega \right)}{T\left(\omega \right)}=\frac{2\pi }{\kappa_{R}}g^{2}N.$$

Importantly, this expression is general for any form and strength of disorder. The final form of this sum rule is similar to the well-established sum rule for the bare molecular system [54]. Note that Eq. (29) is presented in the low temperature limit. For finite temperature effects, see Appendix. Therefore, experimental setups that enable the measurement of both absorption and transmission spectra are deemed ideal for extracting accurate collective light–matter coupling values.

## 3 Conclusions

Our comprehensive investigation has unraveled the intricate relationship between molecular disorder and VRS in molecular polariton. We have derived precise analytical expressions for absorption, transmission, and reflection spectra across various disorder distributions. Furthermore, our study introduces a generalized sum rule for determining the collective light–matter coupling under any form of disorder. These findings do not only clarify the nuanced behavior of VRS amidst disorder but also establish a reliable framework for extracting light–matter coupling parameters with high accuracy from experimental data. In practical terms, when the experimental set-up allows access to both transmission and absorption signals, the sum rule can be readily applied. In situations where accessing both signals is not possible, researchers can leverage Eqs. (2)–(4), coupled with the analytical forms of the molecular susceptibility outlined in Table 1, to effectively fit their experimental results. Both these approaches ensure the extraction of the correct value of $g\sqrt{N}$ in the presence of disorder of any magnitude. Additionally, for scenarios involving mild disorder, the simplified expressions provided in Table 3 offer a convenient solution for data fitting. Furthermore, our study has unveiled a fascinating phenomenon associated with rectangular disorder – the emergence of narrow sidebands alongside a broad central peak. This intriguing line narrowing, observed in the presence of significant disorder, suggests a higher degree of coherence lifetime within the system. Such behavior is especially promising for applications requiring long-lived coherences between the cavity and molecules, providing an exciting avenue for future research in the realm of molecular polaritons.

**Table 3:** Vacuum Rabi splitting expressions for when $\sigma \ll g\sqrt{N}$ .

Optical signal	Vacuum Rabi splitting		
	Gaussian	Lorentzian^a^	Rectangle
*A*(*ω*)	0	$2g\sqrt{N}-\frac{\sigma^{2}}{8\sqrt{g^{2}N-\frac{\kappa^{2}}{8}}}$	0
*T*(*ω*)	$2\sqrt{\frac{1}{2}g^{2}N+\frac{1}{2}\sqrt{g^{4}N^{2}+4g^{2}N\sigma^{2}}}$	$2g\sqrt{N}+\frac{\kappa \sigma }{4g\sqrt{N}}$	$2\sqrt{\frac{1}{2}g^{2}N+\frac{1}{2}\sqrt{g^{4}N^{2}+\frac{\sigma^{2}g^{2}N}{3}}}$
*R*(*ω*)	$2\sqrt{\frac{1}{2}g^{2}N+\frac{1}{2}\sqrt{g^{4}N^{2}+4g^{2}N\sigma^{2}}}$	$2g\sqrt{N}-\frac{\left(\frac{\kappa_{L}^{2}}{\kappa_{R}}-\kappa_{R}\right)\sigma }{8g\sqrt{N}}$	$2\sqrt{\frac{1}{2}g^{2}N+\frac{1}{2}\sqrt{g^{4}N^{2}+\frac{\sigma^{2}g^{2}N}{3}}}$

^a^For larger values of *σ*, Eqs. (16)–(18) should be used to extract the correct value for $g\sqrt{N}$ .

## Appendix A: Sum rule for finite temperature

From Eqs. (2) and (3), we have that

(A1)
$$I=\int d\omega \frac{A\left(\omega \right)}{T\left(\omega \right)}=\frac{\omega_{ph}}{\kappa_{R}}\int d\omega \chi^{\prime \prime }\left(\omega \right)$$

From Eq. (5), for *N* two-level systems coupled to a single cavity mode at a given temperature *T*, Eq. (A1) becomes:

(A2)
$$\begin{array}{ll} I & =\frac{i}{\kappa_{R}}\underset{\gamma \to 0^{+}}{\lim}\overset{N}{\underset{i=1}{\sum }}\tanh\left(\frac{\hbar \omega_{\text{ex},i}}{2k_{B}T}\right)|\lambda \mu_{i}{|}^{2}\\ & \;\times \int d\omega \left[\frac{1}{\omega -\omega_{\text{ex},i}+i\frac{\gamma }{2}}-\frac{1}{\omega -\omega_{\text{ex},i}-i\frac{\gamma }{2}}\right] \end{array}$$

Note that if we evaluate the integral counter clockwise over a semicircle *S*_+_ with an infinite radius in the upper half of the complex plane, we have that

(A3a)
$$\begin{array}{ll} 0 & =\underset{S_{+}}{\oint }\text{d}\omega \left[\frac{1}{\omega -\omega_{\text{ex},i}+i\frac{\gamma }{2}}\right]=\int d\omega \left[\frac{1}{\omega -\omega_{\text{ex},i}+i\frac{\gamma }{2}}\right]+i\pi \end{array}$$

(A3b)
$$\begin{array}{ll} i2\pi & =\underset{S_{+}}{\oint }\text{d}\omega \left[\frac{1}{\omega -\omega_{\text{ex},i}-i\frac{\gamma }{2}}\right]\\ & =\int d\omega \left[\frac{1}{\omega -\omega_{\text{ex},i}-i\frac{\gamma }{2}}\right]+i\pi \end{array}$$

This implies that $\int d\omega \left[\frac{1}{\omega -\omega_{\text{ex},i}+i\frac{\gamma }{2}}\right]=-i\pi$ and $\int d\omega \left[\frac{1}{\omega -\omega_{\text{ex},i}-i\frac{\gamma }{2}}\right]=i\pi$ . Substituting these values into Eq. (A2), we arrive at the generalized sum rule for arbitrary temperature *T*:

(A4)
$$I=\frac{2\pi }{\kappa_{R}}\overset{N}{\underset{i=1}{\sum }}\tanh\left(\frac{\hbar \omega_{\text{ex},i}}{2k_{B}T}\right)|\lambda \mu_{i}{|}^{2}=\frac{2\pi }{\kappa_{R}}Ng_{\text{eff}}^{2}$$

where

(A5)
$$g_{\text{eff}}^{2}=\int d\omega_{\text{ex}}p\left(\omega_{\text{ex}}\right)\tanh\left(\frac{\hbar \omega_{\text{ex}}}{2k_{B}T}\right)|\lambda \mu \left(\omega_{\text{ex}}\right){|}^{2}$$

is the square of the effective single molecule light–matter coupling. Here *p*(*ω*_ex_) is the probability distribution of excitation frequencies. Note in the limit that *ℏω*_ex,*i*_ ≫ *k*_*B*_ *T* and *μ*(*ω*_ex_) → *μ*, we have that $g_{\text{eff}}^{2}\to g^{2}$ , recovering the version of the sum rule presented in the main text.
